# Cross-cultural influences on rhythm processing: reproduction, discrimination, and beat tapping

**DOI:** 10.3389/fpsyg.2015.00366

**Published:** 2015-04-09

**Authors:** Daniel J. Cameron, Jocelyn Bentley, Jessica A. Grahn

**Affiliations:** ^1^Brain and Mind Institute, Western University, London, ON, Canada; ^2^University of Toronto, Toronto, ON, Canada; ^3^Department of Psychology, Western University, London, ON, Canada

**Keywords:** rhythm perception, beat perception, culture, tapping, music

## Abstract

The structures of musical rhythm differ between cultures, despite the fact that the ability to entrain movement to musical rhythm occurs in virtually all individuals across cultures. To measure the influence of culture on rhythm processing, we tested East African and North American adults on perception, production, and beat tapping for rhythms derived from East African and Western music. To assess rhythm perception, participants identified whether pairs of rhythms were the same or different. To assess rhythm production, participants reproduced rhythms after hearing them. To assess beat tapping, participants tapped the beat along with repeated rhythms. We expected that performance in all three tasks would be influenced by the culture of the participant and the culture of the rhythm. Specifically, we predicted that a participant’s ability to discriminate, reproduce, and accurately tap the beat would be better for rhythms from their own culture than for rhythms from another culture. In the rhythm discrimination task, there were no differences in discriminating culturally familiar and unfamiliar rhythms. In the rhythm reproduction task, both groups reproduced East African rhythms more accurately than Western rhythms, but East African participants also showed an effect of cultural familiarity, leading to a significant interaction. In the beat tapping task, participants in both groups tapped the beat more accurately for culturally familiar than for unfamiliar rhythms. Moreover, there were differences between the two participant groups, and between the two types of rhythms, in the metrical level selected for beat tapping. The results demonstrate that culture does influence the processing of musical rhythm. In terms of the function of musical rhythm, our results are consistent with theories that musical rhythm enables synchronization. Musical rhythm may foster musical cultural identity by enabling within-group synchronization to music, perhaps supporting social cohesion.

## Introduction

Music exists in every known culture in history, suggesting that human perception of musical rhythm may be innate and universal (Nettl, 2000). In line with previous work, we define rhythm as a sequence of discrete temporal intervals, marked by (usually auditory) events (Cooper and Meyer, 1960; Fraisse, 1982; Clarke, 1999). In music, rhythms are usually structured such that the time intervals between events are related according to a temporal structure. The universal presence of rhythm may indicate that it has a central, common function. However, rhythmic structures in music vary across cultures, suggesting that culture also influences the perception and production of musical rhythm. Culture encompasses a tremendous range of complex societal constructs, including laws, beliefs, morals, and art. The relevant cultural influences on rhythm likely include, but are not limited to, the auditory experience of music, dance and other types of movement, and language. Despite much ethnomusicological research devoted to identifying and analyzing cultural differences in rhythmic structures, little empirical work has characterized how culture influences human perception and production of musical rhythms.

There may be aspects of rhythm perception that are universal due to common human cognitive processing, and/or physiological dynamics. For example, some work suggests that innate perceptual “rules” govern the perception of accents in temporal groups (e.g., Povel and Essens, 1985), or that resonance in systems of neural oscillations underlie the perception of regularity in musical rhythms (Large and Snyder, 2009). However, experience is known to have an effect on some aspects of rhythm perception. Culture appears to influence rhythm perception as early as 4 months of age: American infants prefer rhythms with a regular metrical structure (found in both Turkish and Western music) to rhythms with an irregular metrical structure (found in Turkish music, but not in Western music). Turkish infants do not have this bias, presumably due to their exposure to music with both regular and irregular metrical structures (Soley and Hannon, 2010). Moreover, both children and adults show superior memory for unfamiliar music from their own culture compared to unfamiliar music from an unfamiliar culture (Morrison et al., 2008). Culture also influences the rhythm of language. Japanese and English speakers differ in their perception of rhythmic tone sequences in ways that are consistent with Japanese and English language rhythms (Iversen et al., 2008). In addition, music and language from a given culture share rhythmic properties. For example, English and French musical rhythmic structures are more similar to English and French speech rhythms (respectively) than to each other, in the sense that English music is more rhythmically variable than French music, and English speech is more rhythmically variable than French speech (Patel et al., 2006). Finally, broader cultural linguistic experience can improve rhythm perception. For example, learning a second language with different rhythmic characteristics than one’s first language improves perceptual discriminability of rhythmic tone sequences (Roncaglia-Denissen et al., 2013). Together, these studies show that enculturation to the rhythmic aspects of music and language occurs early in development and continues into adulthood.

Although it is clear that culture influences rhythm in music and language, the precise aspects of rhythm processing that are influenced by culture are unknown. Few studies have empirically investigated differences in musical rhythm perception between East African and Western music, and between participants from those cultures. As their musical rhythms have distinct characteristics (Temperley, 2000), these cultures are good candidates for comparing rhythm processing. The distinct characteristics of these rhythms lead to differences in perception of meter, the cyclical pattern of strong and weak beats that is perceived in rhythm. Ethnomusicological research on African rhythm has suggested that African music requires greater active engagement in order to maintain meter perception (Chernoff, 1979), puts greater importance on rhythm and meter than Western music does (Chernoff, 1979), commonly has ongoing metrical tension (Agawu, 1995), and tends to be metrically ambiguous (see Temperley, 2000). Metrical ambiguity does not mean that listeners simultaneously perceive more than one meter when listening to a rhythm (e.g., Poudrier and Repp, 2013), but rather that different listeners may perceive different meters in the same musical rhythm. A recurring observation is that in specific cases of African music using cycles of 12 temporal units, African listeners tend to perceive 4 metrical beats of 3 temporal units each (e.g., a 12/8 meter), whereas Western listeners perceive 3 metrical beats of 4 units (e.g., a 3/4 meter; Blacking, 1967; Locke, 1982). In addition to perceptual differences, cultural differences exist regarding aesthetics of rhythm, the evaluation of accuracy in rhythmic performance, and the relative importance of rhythm in music (see Kauffman, 1980; Agawu, 1995). For example, even within cultures, different styles of music might consider notated rhythms to be accurate when they are “swung,” or played “behind the beat.” Thus, we assume that different cultural groups have different notions about what rhythm is or should be, and we account for this in the design of our study.

As East African and Western music differ in their rhythmic structures (Kubik, 1962), we expected that using musical rhythms from these cultures to test participants from each culture (who differ in their exposure to the rhythms) would reveal influences of enculturation on rhythm perception. We assumed that culture, through exposure over time, would influence the processing of rhythm. Therefore, we hypothesized that the culture of the participant and the culture of the rhythmic stimulus would interact in their influence on performance, such that participants would have better performance with rhythms from their own culture. However, because exposure to Western music occurs nearly worldwide, including in the urban setting of our East African sample, our expectations were qualified to consider that both groups would have had exposure to Western musical rhythms, but only East African participants would have had exposure to traditional East African musical rhythms. This is consistent with a study that found that Africans’ and Americans’ ratings of melodic complexity differed for African folk songs, but not for Western folk songs, presumably due to both groups’ familiarity with Western music, but not African music (Eerola et al., 2006).

In addition to our predictions of superior performance (i.e., better ability to discriminate, reproduce, and tap to the beat of rhythms) for culturally familiar rhythms, we expected that culture would influence the range of beat rates that participants tapped. In metrical rhythms, multiple metrical levels (periodicities) are present, and each can legitimately be perceived as the beat. For example, in a 4/4 metrical structure, half notes, quarter notes and eighth notes could each be selected as the beat rate that a listener perceives and thus taps. As African music uses rhythms in which the metrical structure can be interpreted in multiple ways, (i.e., they are metrically ambiguous, see Temperley, 2000), we expected that participants would perceive, and therefore tap to, a greater number metrical levels for East African compared to Western rhythms. In addition, East African participants are assumed to have greater exposure to African music, as well as substantial exposure to Western music, therefore we expected they would tap to a greater number of metrical levels for all rhythms, compared to North American participants. We expected North American participants to tap to fewer metrical levels because their exposure to metrically ambiguous rhythms (such as those found in East African rhythms) is more limited.

Crucially, our *a priori* assumption was that group differences in performance accuracy would *not* be sufficient to demonstrate an influence of culture on rhythm processing. Rather, we would conclude that culture influences rhythm processing only if there was an interaction between the culture of the participant and the culture of the stimulus rhythm. That is, an influence of culture would only be supported if the performance differences between the two types of rhythms also differed between the two groups. A simple group difference would be insufficient because other uncontrolled factors also differed between the groups and may have influenced performance on the tasks. These factors include familiarity with computer-based tasks, language barriers between experimenter and participant, conceptualization of regular beat tapping with auditory rhythms, etc. Therefore, although we observed differences between groups, we cannot identify the specific cause of these differences, and it is the interaction between participant group and rhythm type that we interpreted.

## Materials and Methods

### Participants

Sixteen East African participants were recruited in Kigali, Rwanda (3 female, 23 mean years of age, 3.4 mean years of musical training, 2.5 mean years of dance training). Twenty-five North American participants were recruited in London and Toronto, Canada (13 female, 24.7 mean years of age, 4.7 mean years of musical training, 1.6 mean years of dance training). Musical training included any of the following: private lessons, instrumental or choral experience in school, church, or other organized setting (e.g., regularly performing traditional music ensemble). Dance training included any type of dance, but participants did not have to specify in which types they had training. All participants were over the age of 18, had normal hearing, and had spent the majority of their lives in the respective recruitment regions (East Africa or North America). Age, years of dance training, and years of musical training did not differ significantly between groups, as per independent samples *t*-tests (*p* > 0.05). All participants gave informed consent prior to participating, and were compensated for their participation, as per approval by the ethics boards at the Centre Hospitalier Universitaire de Kigali and the University of Western Ontario.

### Stimuli

**FIGURE 1:**
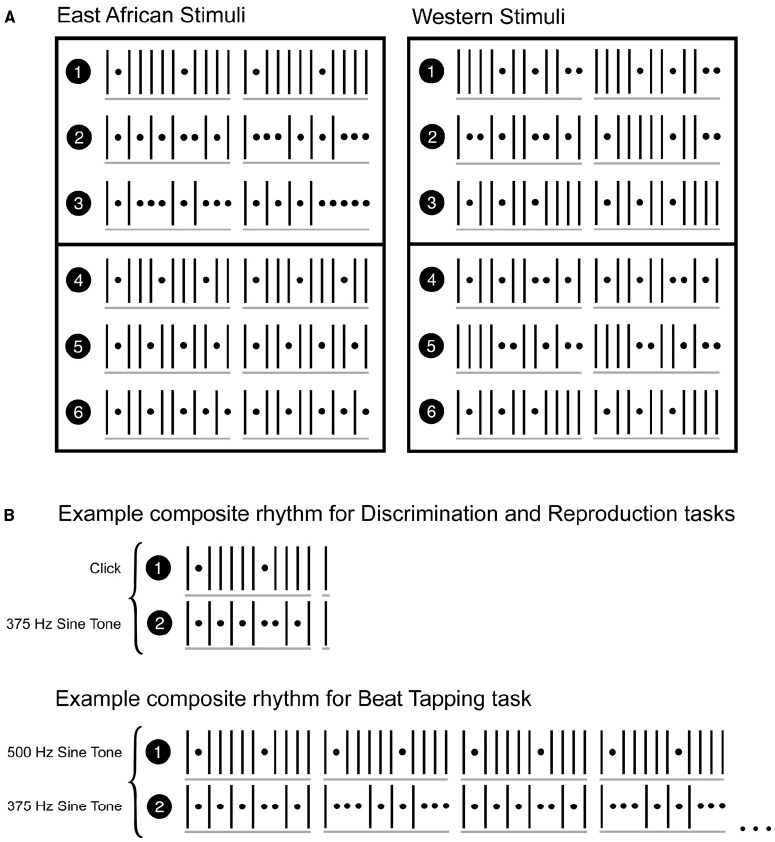
**Stimulus rhythms.** Vertical lines denote onsets, dots denote rests. Each position (onset or rest) is of equal duration, one of 180, 210, or 240 ms. **(A)** Depicts individual rhythms. Rhythms were presented in pairs, as composite rhythms. Both rhythms in a given composite rhythm were selected from the same group of three rhythms (rhythms numbered 1–3, and 4–6). Therefore, within each type (Western and East African), rhythm pairings were 1–2, 1–3, 2–3, 4–5, 4–6, 5–6. For the discrimination and reproduction tasks, only the first cycle of each rhythm (the first 12 units, plus the subsequent downbeat) was used. **(B)** Depicts examples of composite rhythms as used in the tasks. For the beat tapping task, entire rhythms as shown in **(A)** were repeated cyclically (only two repetitions are shown in the example in **B**). Audio examples of the stimuli (one of each rhythm type for each of the tasks) can be found in the supplementary material.

East African rhythms were derived from two recordings of traditional East African music. These were an *embaire* performance called “Muliranwa” by the Ekidha Tobana Kabaliga Group in Bugwere village, Uganda, and an excerpt of a piece called *Chakacha*, performed by the Horizon Players Group and the choir from the Muslim Secondary School in Kisumu, Kenya (Barz, 2004). Three rhythms from each recording were used. The author (DC) transcribed the East African rhythms, and composed the Western rhythms. Western rhythms were composed to conform to norms of Western music in a 12/8 metrical structure, indicating a strong beat on every fourth position in the 12-position cycle. Rhythms were presented as sequences of sine tones or clicks, depending on the task in which they were presented (sine tones and clicks were used for the discrimination and reproduction tasks, and only sine tones were used for the beat tapping task, as described below). Sine tones were 100 ms in duration, had intensity ramped up/down over the first/final 50 ms, and were either 375 or 500 Hz. Clicks were brief (6 ms) excerpts of a generic snare drum sound from audio software (GarageBand). We used synthesized rhythmic tone sequences whose structures were derived from Western and East African music, rather than actual music or recordings of musical instruments, to avoid source-familiarity bias (Van de Vijver and Poortinga, 1997). All rhythms had a temporal structure of 12 units of equal duration; each unit either began with a sound or was silent. Importantly, rhythms were always presented in simultaneous pairs in each trial of each task. This was done to provide more rhythmic and metrical context than individual rhythms could alone, and to thus increase the perceptual differences between East African and Western rhythms. For each cultural rhythm condition, there were six individual rhythms, divided into two groups, each with three individual rhythms. Rhythms were simultaneously presented only with one of the others from the same group, as shown and described in Figure 1. The resulting “composite rhythms” were used as stimuli for all three tasks. For example, the first East African group was composed of rhythms 1–3, and the pairings were: rhythm 1 with rhythm 2, rhythm 1 with rhythm 3, and rhythm 2 with rhythm 3. Because an individual rhythm was created from one of two pitches/sounds, this made for a total of 12 rhythmic stimuli from each culture (e.g., rhythm 1 at 375 Hz with rhythm 2 at 500 Hz, or rhythm 1 at 500 Hz with rhythm 2 at 375 Hz). Each pair of rhythms was used in all tasks. Rhythms could be one of three tempi: each tempo had a unit duration of 180, 210, or 240 ms, respectively. In all trials of all tasks, paired rhythms had the same tempo. Tempo was balanced across conditions in each task. See Figure 1 for a graphical depiction of rhythm stimuli.

For the beat tapping task, the two paired, simultaneously presented rhythms, which together constitute a composite rhythm, were composed of tones of different frequency (pitch), and rhythms were repeated for between 32 and 35s, to give participants enough time to perceive the beat, begin tapping, and stabilize the timing of their taps, as well as provide enough taps for robust measures of variability and accuracy. For the discrimination and reproduction tasks, one of the paired, simultaneously presented rhythms in each composite rhythm, was composed of a sine tone, and the other was composed of the click sound. This was to facilitate distinguishability of the rhythms since the tasks required reproduction or discrimination (from a potentially altered version) of only one of the two rhythms. Examples of stimuli presentation from each task can be found in the Supplementary Material.

All tasks were presented using E-Prime software (Schneider et al., 2002a,b) on a laptop and auditory stimuli were presented via headphones. Trial order was randomized for each task. All responses and tapping were executed on the laptop keyboard.

### Procedure

#### Beat Tapping Task

Participants were instructed to tap the beat of the composite (paired) rhythms. The difference between isochronous beat tapping and non-isochronous rhythm tapping was explained. Participants were asked to listen to the stimulus and, as soon as they felt a sense of the beat, to begin tapping the beat on the “m” key of the laptop keyboard along with the stimulus and to continue until the stimulus stopped. Participants were instructed that their perception of the beat might change over the course of the trial, and that their tapping might naturally adapt to their perception, but to avoid intentionally changing metrical interpretation or beat rate when not induced to by the stimulus (i.e., to not change when they tapped just to make the tapping more interesting). There were 12 trials of each condition for a total of 24 trials, plus two practice trials to begin.

#### Rhythm Discrimination Task

Participants were instructed to listen to three successive presentations of composite rhythms and decide if the third presentation was the same as or different from the first two presentations (which were always identical). During the task, the first composite rhythm was presented twice, accompanied by the text “Original rhythm: First Listen” and “Original rhythm: Second Listen,” and the second composite rhythm was presented only once, accompanied by the text “SECOND rhythm.” Participants were then prompted to make their response by the text “Was the SECOND rhythm the same or different? If same, press (S) and if different, press (D).” Participants responded by pressing keys on the laptop keyboard. Half of the trials in each cultural rhythm condition were “same” and half were “different.” The composite rhythms were always made up of one sine tone sequence and one click sequence, and participants were told that only the tone sequence, not the click sequence, would sometimes contain a change, and only in the third presentation. For “same” trials, all three presentations of the composite rhythms were identical. For “different” trials, the rhythm in the third presentation was altered by switching (transposing) two intervals. This alteration occurred only in the individual rhythm that was composed of tones. The individual rhythm composed of clicks was always the same in the first and second composite rhythm (i.e., it was the same in all three rhythm presentations). There were 12 East African trials and 12 Western trials for a total of 24 trials, plus two practice trials to begin.

#### Rhythm Reproduction Task

Participants were instructed to reproduce a rhythm as accurately as possible after listening to it presented as part of a composite rhythm. They were explicitly instructed to reproduce the rhythms at the same tempo as the presented rhythms. In each trial, a composite rhythm was presented twice, accompanied by the text “rhythm,” followed by a screen signifying the start of the reproduction phase, accompanied by the text “tap back.” Participants tapped the individual rhythm that was presented as a tone sequence in the composite rhythm, on the “m” key of the laptop keyboard. If the rhythm was reproduced accurately, the participant would move on to the next trial. If inaccurate, the participant would attempt the same trial again, up to a maximum of five attempts per trial. Participants each completed 12 trials of each rhythm type for 24 total trials, plus three practice trials. Additionally, participants could repeat the three practice trials if they felt unsure of the task requirements.

Participants in Rwanda underwent EEG recording while listening to stimulus rhythms after completing all three behavioral tasks. These data will be reported elsewhere.

### Analyses

#### Beat Tapping Task

To measure tapping variability, inter-tap intervals (ITIs) were calculated. Individual ITIs were removed if they were less than 0.5 or greater than 1.5 of the mean ITI for that trial. This outlier removal procedure was performed once, then the mean ITI was recalculated and the procedure was performed again. 1.90% of ITIs were removed on this basis. The coefficient of variation (CV) of ITIs was calculated for each trial. The CV was equal to the standard deviation of ITIs divided by the mean ITI for that trial. Trials with a CV greater than 0.2 were removed, as they were considered too variable for the participant to have been intending to tap isochronously. 1.82% of trials were removed on this basis. Additionally, trials with fewer than five taps were removed, and 0.02% of trials were removed on this basis. Participants with five or more trials from each condition removed had their beat tapping data excluded from analyses entirely on the assumption that they did not understand the task requirements or were unable to execute the task consistently. The data from one North American participant was removed on these grounds. Four additional North American participants had no beat tapping data due to technical failure during testing.

To measure tapping accuracy, the absolute asynchrony between each tap and the nearest beat position was calculated. Beat positions occurred at each time point separated by the inter-beat interval (IBI), starting at 0. The IBI was determined by comparing the mean ITI to potential IBIs that were multiples (1, 2, 3, 4, or 6 times) of the tempo. This meant that accuracy could be meaningfully analyzed regardless of what metrical level of the rhythm the participant chose to tap to. The proportional average absolute asynchrony (mean absolute asynchrony divided by the mean ITI) was calculated for each trial to indicate beat tapping accuracy. The metrical level selected by each participant on each trial was determined by finding the multiple of the tempo (1, 2, 3, 4, or 6 times the tempo of 180, 210, or 240 ms, depending on the trial) closest to the mean ITI for that trial. The number of different metrical levels tapped for each rhythm type was calculated for each participant, giving a measure of the tendency of that participant to employ different metrical levels when tapping the beat.

In addition, to measure which of the five metrical levels were tapped to most frequently, for each rhythm type, we calculated the proportion of trials that each metrical level was selected as the beat rate tapped, for each participant.

#### Rhythm Discrimination Task

For the discrimination task, *d′* (sensitivity index) scores were calculated for each participant, for each rhythm type. This statistic measures a participant’s sensitivity to changes in the rhythms, taking into account the participant’s response bias (a bias to respond “same” more often than “different,” or vice versa).

#### Rhythm Reproduction Task

For the rhythm reproduction task, the proportion of trials in which the rhythm was accurately reproduced was calculated for each participant, for each rhythm type. Rhythm reproduction was considered accurate when the correct number of intervals was tapped, and the duration of each tapped interval was within 20% of the presented duration.

For each task, dependent measures were analyzed separately in 2 × 2 mixed analyses of variance (ANOVA) with the between subjects factor of group (East African vs. North American) and the within subjects factor of rhythm type (East African vs. Western). The only exception was for the analysis of which metrical levels were tapped to most frequently in the beat tapping task. For this measure, a 2 × 2 × 5 mixed analysis of variance was used, with the same two factors (group and rhythm type) and the repeated measures factor of metrical level (intervals of 1, 2, 3, 4, or 6 times the tempo). In cases where the assumption of sphericity was violated, Greenhouse–Geisser corrections were applied. Follow up *t*-tests were completed to investigate differences between individual conditions, in the case of significant interactions of group and rhythm type.

## Results

### Beat Tapping Task

**FIGURE 2:**
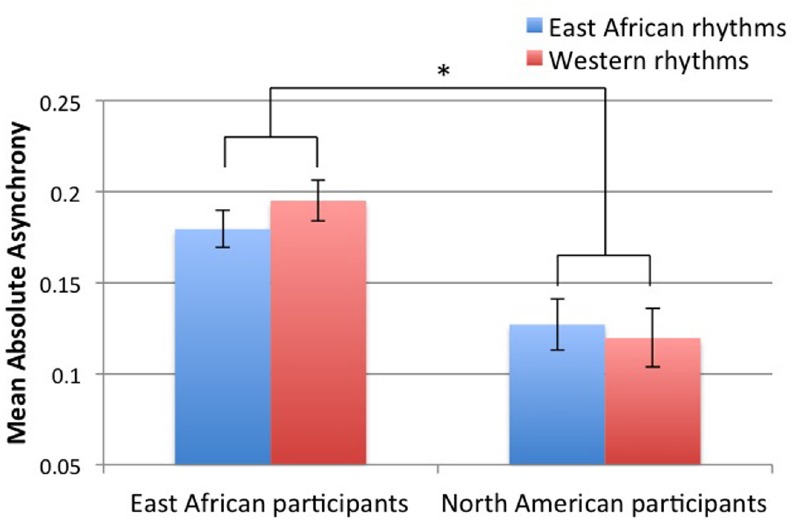
**Absolute asynchrony values of beat taps relative to beat positions in the rhythmic stimuli, averaged over each trial and proportionate (divided by) the tapping rate (mean ITI).** Error bars indicate (±1 SE of the mean. *p < 0.05 (interaction between group and rhythm type).

Tapping variability (CV of ITI) did not significantly differ for rhythm type or group, and there was no significant interaction between those factors. However, North Americans tapped to the beat with greater accuracy (lower mean asynchrony) than East African participants [main effect of group: *F*(1,34) = 11.29, *p* = 0.002]. There was also an interaction between group and rhythm type: each group tapped more accurately to the beat of rhythms derived from music of their respective culture [*F*(1,34) = 3.48, *p* = 0.071] as shown in Figure 2. Although the *p* value of this *F* test is not below 0.05, it is below 0.1, and we interpret this result because the direction of differences was predicted (thus, the equivalent of a one-tailed probability test is justified, and the *p* value can be reported as 0.036).

**FIGURE 3:**
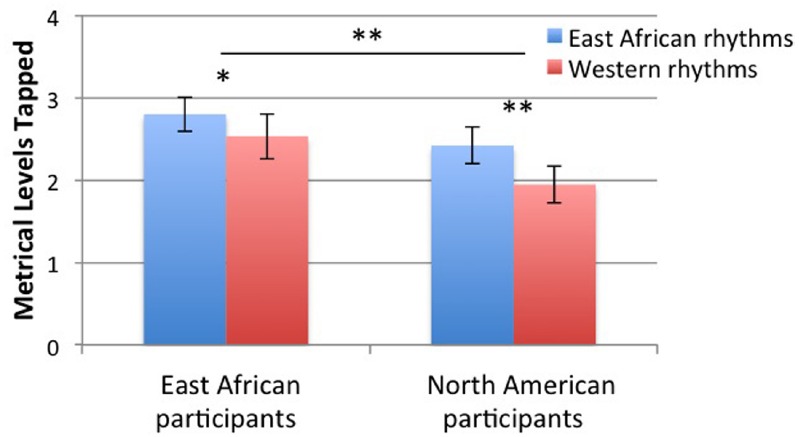
**Number of metrical levels tapped.** Error bars indicate ±1 SE of the mean. *p < 0.1, **p < 0.05.

Participants tapped to a greater number of metrical levels for East African rhythms than for Western rhythms [main effect of rhythm type: *F*(1,34) = 7.13, *p* = 0.011], and East African participants tapped to a greater number of metrical levels than North American participants [main effect of group: *F*(1,34) = 3.11, *p* = 0.087], as shown in Figure 3. We interpret the main effect of group, despite a *p* value over 0.05, for the same reason described above: the *p* value of this *F* test is between 0.05 and 0.1, and the direction of differences was predicted (thus, the equivalent of a one-tailed probability test is justified, and the *p* value can be reported as 0.044). The two factors did not interact.

**FIGURE 4:**
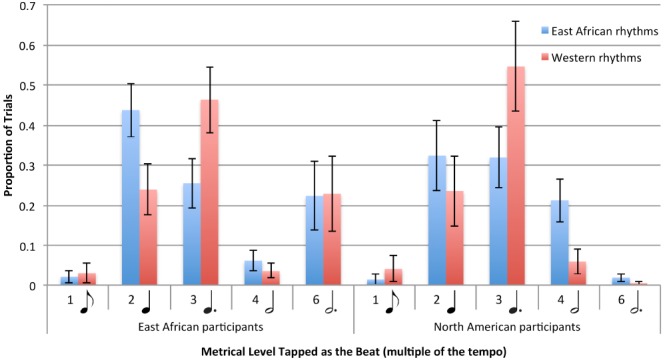
**Proportions of different metrical levels tapped as the beat for East African and Western rhythms by East African and North American participants.** Metrical levels are multiples of the tempo (the tempo in turn is the duration of the unit equal to an eighth note in a 12/8 metrical structure, thus, higher metrical levels are slower beat rates). Error bars indicate ±1 SE. of the mean.

Participants across both groups selected certain metrical levels to tap to more often than others, irrespective of the type of rhythm [main effect of metrical level: *F*(1,31) = 5.57, *p* = 0.004]. However, the proportion of trials tapped at each metrical level differed between East African and Western rhythms [interaction between metrical level and rhythm type: *F*(1,31) = 8.99, *p* < 0.001], as shown in Figure 4. Participants selected the third metrical level more often for Western than East African rhythms [*t*(31) = 5.04, *p* < 0.001], and the second and fourth metrical levels more often for East African than Western rhythms [second metrical level: *t*(31) = 3.79, *p* < 0.001; fourth metrical level: *t*(31) = 3.22, *p* = 0.003]. There was no indication that the two groups significantly differed in their use of metrical levels over others for the two types of rhythms [interaction between metrical level and group: *F*(1,31) = 2.05, *p* = 0.122], or that the difference in proportion of metrical levels selected between the two types of rhythms differed between groups [interaction between metrical level, group, and rhythm type: *F*(1,31) = 1.82, *p* = 0.173].

### Rhythm Discrimination Task

**FIGURE 5:**
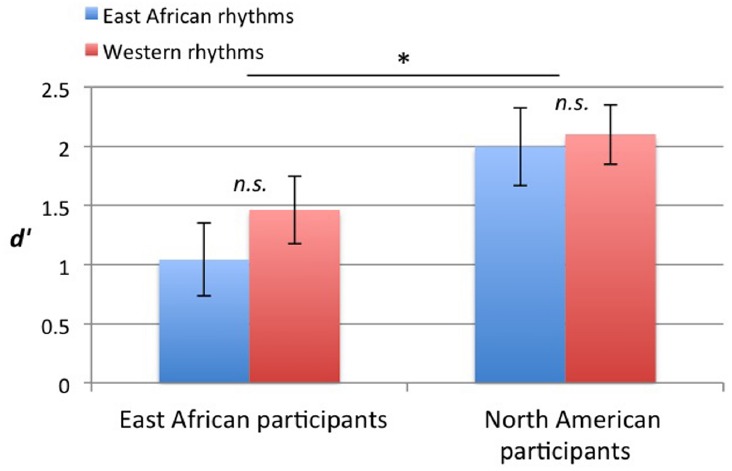
***d′* scores for the discrimination task, reflecting accuracy in discriminating rhythms.** Error bars indicate ±1 SE of the mean. *p < 0.05.

North American participants discriminated rhythms more accurately than East African participants [main effect of group: *F*(1,37) = 4.53, *p* = 0.040], but there were no main effects of rhythm type, nor interaction between group and rhythm type, as shown in Figure 5.

### Rhythm Reproduction Task

**FIGURE 6:**
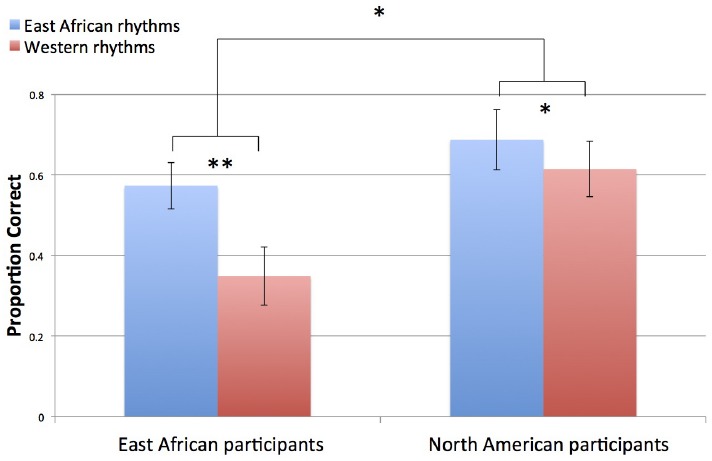
**Proportion of accurately reproduced rhythms.** Error bars indicate ±1 SE of the mean. *p < 0.05, **p < 0.01.

East African rhythms were reproduced more accurately than Western rhythms [main effect of rhythm type: *F*(1,38) = 18.00, *p* < 0.001], and there was a marginally significant effect of group, suggesting that North American participants reproduced more rhythms accurately than East African participants did [main effect of group: *F*(1,38) = 3.63, *p* = 0.064] However, there was also a significant interaction between group and rhythm type [*F*(1,38) = 5.5, *p* = 0.024]. Paired *t*-tests showed that both groups accurately reproduced a greater proportion of East African rhythms than Western rhythms (East African participants: *t*(15) = 4.02, *p* = 0.001; Western participants: *t*(23) = 2.45, *p* = 0.023). Independent samples *t*-tests showed that Western participants were better than East African participants at reproducing Western rhythms [*t*(38) *=* 2.59, *p* = 0.014] but that the groups did not differ in proportion of accurately reproduced East African rhythms [*t*(38) *=* 1.22, *p* = 0.273], as shown in Figure 6.

## Discussion

Overall, we find evidence of culture’s influence on rhythm perception, rhythm production, and beat tapping. As predicted, culture influenced performance on the beat tapping and rhythm reproduction tasks. Culture may influence rhythm perception by both active engagement with, and passive exposure to music, over time. The finding of cultural influence is consistent with past work suggesting rhythm perception is malleable by culture rather than innate and universal, and extends beyond that work by testing adults on multiple tasks using rhythms from two cultures with distinct musical rhythms.

Although North American participants generally performed better than East African participants, there were differences in the testing conditions between groups that prevent interpretation of group differences. Most notably, language and cultural barriers were present between participants and the experimenter for the East African group but not the North American group. Moreover, many North American participants were familiar with typical behavioral psychology experiments, instructions, testing environments, and equipment, which potentially biases the tests toward that group. Therefore, group differences in task performance may reflect differences in response to the testing conditions, rather than true cultural differences in rhythm and beat perception ability. As mentioned above, our *a priori* assumption was that a group difference in performance accuracy would not constitute evidence of an influence of culture. However, group differences in the *nature* of performance within a task (i.e., for the number of metrical levels tapped in the beat tapping task) are interpretable because that measure would not be sensitive to familiarity with the task instructions, environment, and equipment, and was predicted to differ between groups. Similarly, differences in task performance between the rhythm types, and interactions between group and rhythm type, are valid, as they are within-subject factors and thus resilient to testing biases between groups.

### Beat Tapping Task

The results indicate that culture influences beat tapping accuracy. Participants from both groups tended to tap the beat with greater accuracy when tapping with rhythms derived from music of their own culture. Interestingly, tapping variability was not influenced by culture. Cultural familiarity may therefore benefit the precision of identifying and anticipating beat positions in a rhythm, but not the ability to maintain a steady tapping rate. Another possibility is that the ability to tap steadily varies more across individuals than it does across cultures, in which case our measures may not have been sufficiently sensitive to demonstrate an effect.

Metrical interpretation varied across rhythm type as well as across group. As predicted, participants tapped to a greater number of metrical levels for East African rhythms than Western rhythms, presumably because East African rhythms allow more options for metrical interpretation than Western rhythms. Also as predicted, East African participants tapped to more metrical levels than Western participants, presumably due to their greater exposure to music containing rhythms that allow flexible metrical interpretations. East African participants would have had more opportunities to hear music with these rhythms, and moreover, to move to them (e.g., through dancing or clapping). This experience may have transferred to the simpler tapping movements required by the task.

In addition to analyzing the number of different metrical levels that were tapped across conditions, we also examined which of those metrical levels were tapped to most often across conditions. The specific metrical levels that participants chose to tap as the beat differed between East African vs. Western rhythms. Participants selected the third metrical level (a dotted quarter note in a 12/8 time signature) more often when tapping the beat with Western rhythms than East African rhythms. They selected the second and fourth (quarter note and half note) more often for East African than Western rhythms. These differences between tapping with East African and Western rhythms were significant, and presumably related to the different structural characteristics of the two types of rhythms. However, comparisons between the two groups (participants from East Africa and North America) did not reach significance, although, in terms of absolute proportions, East African participants tapped more often at the highest metrical level, a dotted half note in a 12/8 meter, than North American participants (see Figure 4). Overall, the data do not refute the null hypothesis that the groups do not differ in the metrical levels they select to tap to in the rhythms. Given the suggestions of ethnomusicological work, it may be that our study lacked sufficient power to demonstrate these differences (the *p* value for the group by metrical level interaction was 0.12).

### Rhythm Reproduction Task

An influence of culture was also found for rhythm reproduction accuracy. Both groups reproduced East African rhythms more accurately than Western rhythms, but the difference between rhythm types was larger for the East African group than the North American group. This can be interpreted in two ways: East African rhythms were easier to reproduce overall, and the advantage of tapping those rhythms compared to Western rhythms was greater for East African participants than for North American participants. This suggests that East African participants benefitted from their cultural familiarity with East African rhythms. Another interpretation is that the Western rhythms were more difficult for East African participants than for Western participants, but that both groups found East African rhythms similarly easy. This suggests that tapping the culturally unfamiliar compared to familiar rhythms was more difficult for East African participants. In either case, the results are consistent with the prediction that cultural exposure to musical rhythms facilitates the reproduction of those rhythms.

### Rhythm Discrimination Task

No influence of culture was found on accuracy for identifying whether a rhythm was the same as or different from another rhythm. Although the groups performed differently, this may be attributable to factors other than of rhythm perception differences, such as differences in familiarity with computer-based tasks and behavioral testing situations. There may be no true effect of culture on rhythm discrimination, or the task and stimuli may not have been optimal for detecting cultural influences on this perceptual task. The alteration of the rhythms for the “different” trials in the discrimination task may have made the rhythms musically implausible, thus reducing the effect of cultural exposure to music. A lack of detection of a real effect is plausible, as previous studies have used purely perceptual measures of rhythm to demonstrate the influence of culture (e.g., Eerola et al., 2006; Morrison et al., 2008; Soley and Hannon, 2010).

### General Discussion

Our findings demonstrate that culture can influence the processing of musical rhythm and beat. If we assume that familiarity (e.g., as gained by cultural exposure) enhances performance generally, then the sensitivity of rhythm and beat production to culture are consistent with a function of musical rhythm being to facilitate synchronization. Musical rhythm may support cultural identity because it can facilitate interpersonal synchrony, consistent with theories that the function (i.e., adaptive value) of music and musical rhythm is to facilitate social cohesion (Huron, 2001). However, it is important to note that the function(s) of music may differ between cultures. Previous work (Jones, 1959; Blacking, 1967; Chernoff, 1979; Locke, 1982; Agawu, 1995), and the discipline of ethnomusicology, generally, provides greater detail and insight into the sociocultural contexts and functions of African and Western music.

Testing the influence of culture is challenged by the need for stimuli that avoid information that provide other musical context (i.e., stimuli that are well controlled, so that effects can be attributed to the differences in rhythm), but also accurately reflect the broader musical context from which rhythms were drawn and exert influence through exposure (i.e., stimuli that are ecologically valid, so that a real effect of culture can be detected). In this study, rhythmic stimuli consisted of synthesized tone sequences rather than real music or sounds from musical instruments in order to maintain control, and also consisted of paired, overlapping rhythms in order to create a musically realistic context. It is possible that our choices of rhythms were not ideal for demonstrating a cultural influence on rhythm perception (e.g., due to a lack of sufficient cultural familiarity with the rhythms), or that other cultures have more distinct musical rhythms. Future studies may yield more sensitivity to the influence of culture on rhythm processing by comparing rhythmic stimuli and participants from cultures with more distinct musical rhythms.

Future studies could also combine cross-cultural approaches with neuroimaging methods to better understand the neural mechanisms of rhythm perception. In one neuroimaging (fMRI) study, no differences were found in neural activations while listening to culturally familiar vs. culturally foreign music, despite the fact that music culture influenced performance on a recall task (Morrison et al., 2003). However, another approach could use cultural differences and fMRI to better understand rhythm perception: since most fMRI studies of rhythm have used Western rhythms and participants, having non-Western participants listen to rhythms perceived as irregular by Western participants but regular by non-Western participants, could reveal activations in either the same or different networks found for rhythm and beat perception in past studies. This approach could help elucidate the role of familiarity and regularity in the neural mechanisms of rhythm perception.

To conclude, this study provides empirical support for an enhancing influence of culture on the perception and production of musical rhythm. Future studies could build on this work to investigate the cultural influence on neural mechanisms of rhythm and beat perception, and whether there are aspects of rhythm processing *not* influenced by culture (i.e., are universal).

### Conflict of Interest Statement

The Guest Associate Editor Fabien Gouyon declares that, despite having collaborated on the Research Topic (Functions of musical rhythm) with author Jessica A. Grahn, the review process was handled objectively and no conflict of interest exists. The authors declare that the research was conducted in the absence of any commercial or financial relationships that could be construed as a potential conflict of interest.
